# Antimicrobial Activities of Alginate and Chitosan Oligosaccharides Against *Staphylococcus aureus* and Group B *Streptococcus*

**DOI:** 10.3389/fmicb.2021.700605

**Published:** 2021-09-13

**Authors:** Mostafa Asadpoor, Georgia-Nefeli Ithakisiou, Jos P. M. van Putten, Roland J. Pieters, Gert Folkerts, Saskia Braber

**Affiliations:** ^1^Division of Pharmacology, Faculty of Science, Utrecht Institute for Pharmaceutical Sciences, Utrecht University, Utrecht, Netherlands; ^2^Division of Infectious Diseases and Immunology, Department of Biomolecular Health Sciences, Faculty of Veterinary Medicine, Utrecht University, Utrecht, Netherlands; ^3^Division of Medicinal Chemistry and Chemical Biology, Faculty of Science, Utrecht Institute for Pharmaceutical Sciences, Utrecht University, Utrecht, Netherlands

**Keywords:** alginate oligosaccharides, chitosan oligosaccharides, *Staphylococcus aureus*, group B *Streptococcus*, bacterial growth, anti-biofilm, synergy, sensitization

## Abstract

The bacterial pathogens *Streptococcus agalactiae* (GBS) and *Staphylococcus aureus* (*S. aureus*) cause serious infections in humans and animals. The emergence of antibiotic-resistant isolates and bacterial biofilm formation entails the urge of novel treatment strategies. Recently, there is a profound scientific interest in the capabilities of non-digestible oligosaccharides as antimicrobial and anti-biofilm agents as well as adjuvants in antibiotic combination therapies. In this study, we investigated the potential of alginate oligosaccharides (AOS) and chitosan oligosaccharides (COS) as alternative for, or in combination with antibiotic treatment. AOS (2–16%) significantly decreased GBS V growth by determining the minimum inhibitory concentration. Both AOS (8 and 16%) and COS (2–16%) were able to prevent biofilm formation by *S. aureus* wood 46. A checkerboard biofilm formation assay demonstrated a synergistic effect of COS and clindamycin on the *S. aureus* biofilm formation, while AOS (2 and 4%) were found to sensitize GBS V to trimethoprim. In conclusion, AOS and COS affect the growth of GBS V and *S. aureus* wood 46 and can function as anti-biofilm agents. The promising effects of AOS and COS in combination with different antibiotics may offer new opportunities to combat antimicrobial resistance.

## Introduction

Among various pathogenic agents, *Staphylococcus aureus* (*S. aureus*) and Group B *Streptococcus* (GBS), alternatively called as *Streptococcus agalactiae* (*S. agalactiae*), cause serious infections in both humans and animals at a global scale. These pathogens can cause a wide spectrum of invasive diseases ranging from neonatal sepsis, meningitis, and pneumonia to severe mastitis in cattle (Tong et al., 2015; Lin et al., 2017). Both pathogens produce multiple virulence factors and have the capability to form biofilms (Rosini and Margarit, 2015; Moormeier and Bayles, 2017). An increasing problem in treating these infections is the emergence of strains that are resistant to antimicrobial treatment (Costerton et al., 1999; Jamal et al., 2018). Infections related to the pathogenic form of GBS might occur *in utero* or with its passage through the birth canal during parturition. The percentage of neonates from GBS-colonized mothers that become transiently colonized with GBS by their mother’s organism is about 30–70% (Melin, 2011). Despite its role as a common intestinal colonizer in infants, how GBS retains its potential virulence and its transition from a commensal to a devastating pathogen remains poorly understood (Kolter and Henneke, 2017). On the other hand, *S. aureus* causes a wide range of diseases, such as toxic shock syndrome, infective endocarditis, as it is capable to disrupt tissue barriers, entering the bloodstream, and contaminating almost every organ in the body (Brandt et al., 2018). Additionally, *S. aureus* is the most common causative pathogen of infectious mastitis that might appear at every stage of life but occurs mostly in women during the breast-feeding period. The global incidence of mastitis within lactating women varies from 1 to 10% although some studies indicate that this infection can be observed to reach 33% of lactating women (Boakes et al., 2018). Through breastfeeding, *S. aureus* can be transferred to the gut microflora of newborns, where it colonizes the gastrointestinal tract (Lindberg et al., 2004).

The high prevalence of pediatric infectious diseases linked with the colonization, and the pathogenicity of GBS and *S. aureus* in the gastrointestinal tract of infants has increased the attention toward alternative approaches to prevent/reduce the incidence of these infections. Misuse and overuse of broad-spectrum antibiotics has resulted in a situation, wherein bacteria promote the evolution of phenotypes resistant to nearly every antibiotic in clinical use (Craft et al., 2018a). Therefore, there is an urgent need for alternatives that can tackle the problem of antimicrobial resistance. Antibiotic combination therapy, which involves the co-administration of antibiotics with an adjuvant that suppresses the resistance and enhances the antibiotic function and efficacy, offers promising therapeutic perspectives (Wright, 2016). One of the mechanisms that bacteria used to develop resistance to antibiotics is based on the alteration of their physiology through the formation of a biofilm matrix. It is estimated that 65% of all bacterial infections result in bacterial biofilm formation (Jamal et al., 2018), one of the interesting characteristics of many bacteria, including *S. aureus* and GBS (Rosini and Margarit, 2015; Moormeier and Bayles, 2017). Especially, biofilm formation on implanted materials and medical devices, such as catheters, endotracheal tubes, and prosthetic joints, poses a serious public health problem (Jamal et al., 2018).

The composition and the stability of biofilms is dependent on the structure of their extracellular polymeric substances (EPS), a matrix that is mainly composed of polysaccharides, proteins, lipids, and extracellular DNA (eDNA; Flemming et al., 2016). Different hypotheses have been examined to explain the antimicrobial persistence in the unbreakable structures of biofilms. First, it is believed that antibiotics can be inactivated by antibiotic-degrading enzymes, which are accumulated in the biofilm matrix. Second, the low metabolic activity of microorganisms observed in a biofilm is correlated with antibiotic tolerance (Reffuveille et al., 2017). Additionally, the intrinsic structure of biofilms may prevent the antibiotics to penetrate into the biofilm due to a high abundance of water channels (Bjarnsholt et al., 2013; Reffuveille et al., 2017). Given the complex mechanisms responsible for antibiotic resistance of bacteria in biofilms, a combination of various defensive ways may be needed to successfully combat these bacterial structures.

Non-digestible oligosaccharides (NDOs), complex carbohydrates that resist hydrolysis by salivary and intestinal digestive enzymes and known for their prebiotic properties by stimulating beneficial bacteria in the gut microbiota, These NDOs also exhibit various pathogen reduction capabilities, as reviewed in Asadpoor et al. (2020, 2021a), and thus may represent potential therapeutic candidates against infections. NDOs, such as human milk oligosaccharides (HMOs), chitosan oligosaccharides (COS), and alginate oligosaccharides (AOS), two NDOs that structurally resemble HMOs, can exhibit anti-biofilm activity (Ackerman et al., 2018; Powell et al., 2018; Asadpoor et al., 2021a), not only by preventing biofilm formation but also by decomposing preformed biofilms probably *via* the disruption of EPS components (Powell et al., 2018). Furthermore, specific NDOs, and especially HMOs, exert a bacteriostatic effect on bacterial growth (Craft et al., 2018b; Asadpoor et al., 2020). Based on different investigations, AOS and COS have already shown anti-virulence and anti-biofilm properties against different bacteria, such as *Acinetobacter baumannii* and *Pseudomonas aeruginosa* (Khan et al., 2012; Lu et al., 2014).

Given the antimicrobial capacity of AOS and COS, the current study investigated the potential of these two promising NDOs to inhibit bacterial growth and biofilm formation of *S. aureus* and GBS. In addition, the effect of the NDOs in combination with antibiotics was tested to evaluate possible synergistic effects that may diminish the bacterial resistance to antimicrobials.

## Materials and Methods

### Bacterial Strains and Culture Conditions

*S. aureus* strain wood 46 (ATCC 10832; de Vor et al., 2021) and wild-type (WT) GBS clinical isolate NCTC 10/84 (1169-NT1; ATCC 49447; serotype V; Sheen et al., 2011) were gifts from Suzan Rooijakkers (UMC, University Medical Center, Utrecht, Netherlands) and Nina van Sorge (AMC, AcademicMedical Center, Amsterdam, Netherlands), respectively. Bacteria were stored at −80°C. For all experiments, bacteria were first grown on blood agar plates (Biotrading, Mijdrecht, Netherlands) at 37°C for 24h and subsequently, the colonies were sub-cultured in tryptic soy broth (TSB) and incubated overnight at 37°C under shaking conditions (160rpm). After incubation, bacterial growth (OD_600_) was measured and bacterial density was adjusted according to OD600=0.5 (determination of the minimum inhibitory concentration) or at OD600=1 (biofilm formation assay).

### AOS and COS (NDOs)

**Figure 1:**
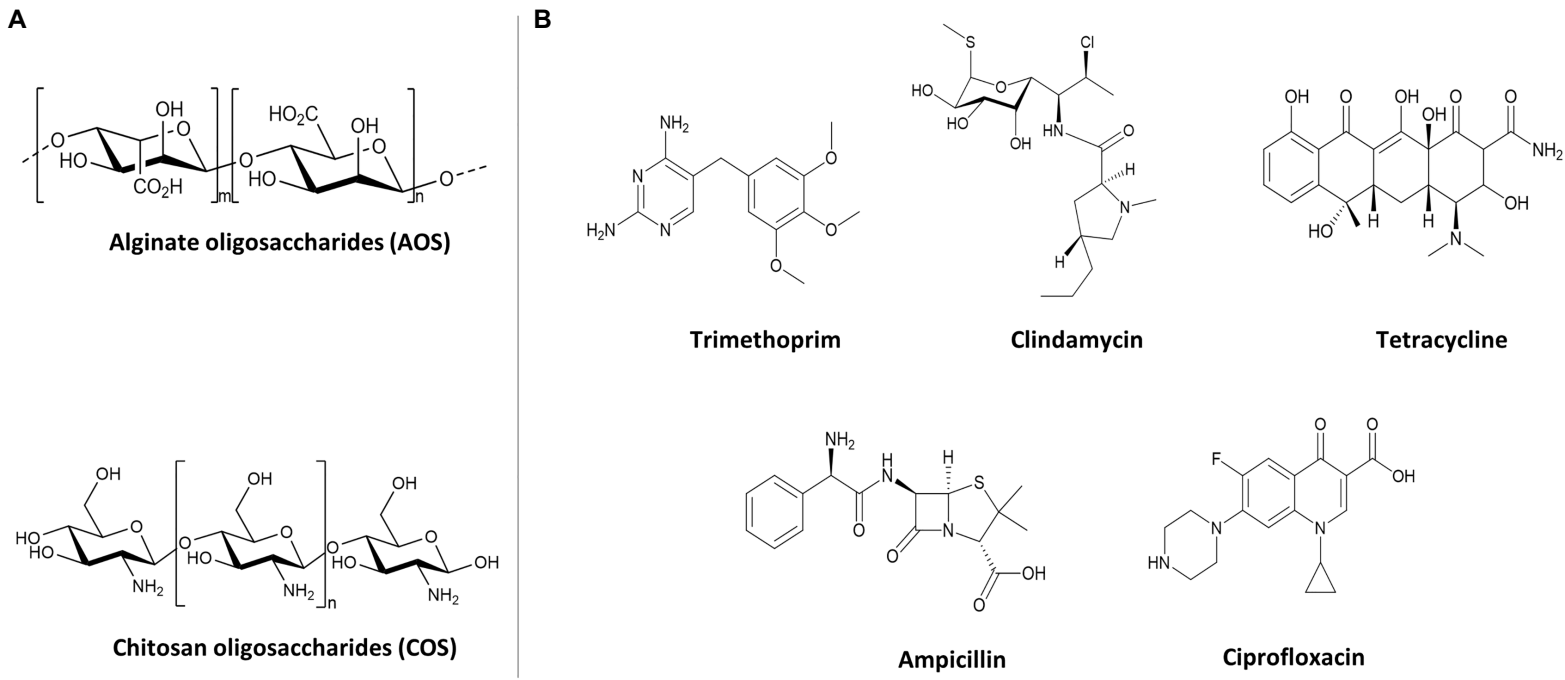
Structures of oligosaccharides and antibiotics used for antimicrobial activity against GBS V and *S. aureus* wood 46. **(A)** Structures of AOS and COS. **(B)** Structures of bacteriostatic (TMP, CLI, and TET) and bactericidal (AMP and CIP) antibiotics.

The NDOs, AOS (purity>85%) and COS (purity>90%), were purchased from BZ Oligo Biotech Co., Ltd. (Qingdao, Shandong, China). AOS was produced by the degradation of algin and COS originated from marine biological sources (shrimp and crab shells). Their structures are depicted in Figure 1A. NDO (AOS and COS) solutions were freshly dissolved in TSB before each experiment and the pH of the solution was adjusted to 7.2–7.4. The chemical NDO structures were drawn using ChemDraw Professional 15.0.

### Antibiotics

Ampicillin (AMP), Ciprofloxacin (CIP), Clindamycin (CLI), Tetracycline (TET), and Trimethoprim (TMP) were purchased from Sigma-Aldrich (Steinheim, Germany). These five common-used antibiotics were chosen based on a variety of chemical structures (Figure 1B) and mechanisms of action to assess whether in combination with AOS and COS can sensitize bacterial strains (*S. aureus* strain wood 46 and GBS V) to these antibiotics. Sterile stock concentrations of each antibiotic were made in TSB and used for serial dilutions and prior to each experiment fresh stocks were prepared. The chemical antibiotic structures were drawn using ChemDraw Professional 15.0.

### Determination of the Minimum Inhibitory Concentration

The antibacterial capacity of AOS and COS against GBS V and *S. aureus* wood 46 was determined *via* analyzing the minimum inhibitory concentration following the method as described previously (Asadpoor et al., 2021b). AOS and COS were serially diluted in 96-well U-bottom polypropylene plates (Corning Costar, Cambridge, MA, United States) to reach 100μl final volume with concentrations ranging from 16 to 0.25%. Subsequently, 100μl of bacterial inoculums (*S. aureus* strain wood 46 and GBS V) with OD600=0.5 (approximately 10^+8^colony forming units [CFU]/ml) were added to serially diluted NDOs (Uriarte et al., 2017; Pinna et al., 2020). The plates were covered with sterile breathable film (VWR International, Amsterdam, Netherlands) and incubated overnight at 37°C under shaking conditions (160rpm). After the incubation period, 100μl of culture medium was transferred to 96-well F-bottom polystyrene microtiter plates (Corning Costar, Cambridge, MA, United States) and the signal was measured at 600nm with a FLUOstar Omega microplate reader (BMG Labtech GmbH, Ortenberg, Germany). Bacteria growth in TSB without treatment served as positive control and TSB alone was used as negative control. The minimum inhibitory concentration was considered as the lowest concentration that inhibits bacterial growth by more than 90% in comparison to positive control groups, as IC_90_ value correlated well with the minimum inhibitory concentration of a compound as described by Sun et al. (2016) and Zheng et al. (2018).

### Antibiotic Sensitization Assay

The ability of AOS to sensitize the GBS strain V to specific antibiotics was determined using the antibiotic sensitization assay as described before (Marks et al., 2013). AOS were serially diluted in 96-well U-bottom polypropylene plates with concentrations ranging from 16 to 2%. Subsequently, AOS was combined with different concentrations of AMP, CLI, TET, and TMP. The concentration range of the studied antibiotics was chosen based on the determination of the minimum inhibitory concentration. Concentrations below the minimum effective concentration were selected in order to create a wide range, in which the additional effect of AOS could be visible. Thereafter, 100μl bacterial inoculums with OD=0.5 was added to the treatments. Plates were covered with sterile breathable film (VWR International, Amsterdam, Netherlands) and incubated for 24h at 37°C under shaking conditions (160rpm). After the incubation period, the optical density was measured with a FLUOstar Omega microplate at 600nm. Bacteria growth in TSB without any treatment served as positive control, while wells with TSB (no treatment) were considered as negative control. For determining statistical significance, the outcome of the combinational treatment was compared to both the results of corresponding antibiotic concentration and corresponding AOS concentration. Therefore, the final effect was considered statistically significant when all two conditions were significant different compared to the combination therapy, suggesting sensitization was achieved.

### Biofilm Formation Assay

The biofilm formation assay was performed for the evaluation of the effect of AOS and COS on the biofilms produced by *S. aureus* wood 46 and GBS V. As described above, for the biofilm formation assay, bacteria strains were grown in TSB at 37°C under shaking conditions (160rpm). Afterward, different procedures were carried out for the growth of streptococci and staphylococci biofilms.

For the development of streptococci biofilm, a biofilm formation assay was adapted from methods described previously (Ruppen et al., 2017). Briefly, NDO treatments of serially diluted concentrations (16–0.5%) were prepared in 96-well F-bottom polystyrene microtiter plates. For the preparation of the serial dilutions of AOS and COS, the biofilm medium (BM) composed of TSB supplemented with 0.5% glucose and 3% NaCl was used. Thereafter, the optical density of GBS V was adjusted at OD600=0.5 and 100μl of the inoculated medium was transferred into the 96-well plates in the presence of increasing NDO concentrations. The biofilms were grown for 24h at 37°C with 5% CO_2_ under static conditions. Furthermore, bacteria grown in BM in the absence of any intervention served as positive control, representing the maximum biofilm growth. Uninoculated culture media (BM: TSB, 1:1) was considered as negative control.

For the biofilm formation by staphylococci, a different procedure was followed as described by Kang et al. (2019). Briefly, the OD of grown bacteria was adjusted at OD600=1. To prepare the working bacterial solution (WBS), 10μl of bacterial solution was added to 10ml of BM (1:1000). NDO treatments were prepared in 96-well F-bottom plates of serial diluted concentrations (16–0.5%). Thereafter, 100μl WBS was added in 96-well F-bottom plates in absence or presence of NDOs and incubated for 24h at 37°C with 5% CO_2_ under static conditions. For quantifying the biofilm inhibitory effect of NDOs, full-formed biofilms without any additional treatment were used as positive control and uninoculated culture media (BM: TSB, 1:1) was used as negative control.

Subsequently, supernatants of both streptococci and staphylococci biofilms were gently removed, wells were washed with 200μl of phosphate-buffered saline (PBS), and the bacterial biofilms were fixed at 60°C for 30min. The fixed biofilms were stained with 160μl of crystal violet (CV) solution (0.1%) for 5min. Excess stain was discarded and wells were washed twice with tap water. Stained biofilms were solubilized in 160μl of acetic acid (33%), and 100μl was gently transferred to a 96-well F-bottom plate. The biofilm formation was measured at 595nm using a FLUOstar Omega microplate reader.

Minimum biofilm inhibitory concentration (MBIC) of NDOs against GBS V and *S. aureus* wood 46 was determined as the lowest concentration of NDOs that inhibit biofilm formation by more than 90% in comparison to control groups (Sun et al., 2016; Zheng et al., 2018).

### Checkerboard Biofilm Formation Assay

The checkerboard biofilm formation assay was performed in order to identify the type of interaction (synergistic, additive, indifferent, or antagonistic) between AOS and COS with the five antibiotics against bacterial biofilm formation by *S. aureus* (Mataraci and Dosler, 2012). Concentration ranges of antibiotics are depicted in Table 1.

**Table 1:** Minimum biofilm inhibitory concentration (MBIC) of *S. aureus* wood 46 and ranges of antibiotic solutions performed in the checkerboard biofilm formation assay.

Antibiotics	MBIC (μg/ml)	Ranges of tested concentrations (μg/ml)
AMP	0.0156	0.0128–0.0002
CIP	0.5	4–0.0078
CLI	0.25	2–0.0039
TET	0.25	2–0.0039
TMP	2	8–0.0156

Serial dilutions of NDOs were prepared with concentrations ranging from 16 to 0.25% and were added to the different rows of the flatbottom 96 well plate. The antibiotic dilutions were added to the different columns of the 96 well plate. In this regard, a variety of mixtures of different concentrations of the assessed compounds (NDOs and antibiotics) was created with controls for NDOs (far-right column) and antibiotics (bottom row). The well at the bottom right corner was used as positive control, in which only inoculated medium was present in order to indicate the maximum biofilm growth. Subsequently, biofilms were prepared and biofilm formation was measured as described in section Antibiotic Sensitization Assay. Briefly, 100μl of the WBS was added to the combined treatments to reach 200μl final volume per well. The plate was incubated at 37°C with 5% CO_2_ for 24h at static conditions. Following the incubation period, the medium was gently aspirated and the wells were washed once with 200μl PBS to remove free-floating “planktonic” bacteria. After the washing procedure, the biofilm was fixed at 60°C for 30min. For measuring the biofilm biomass, the fixed biofilm was stained with 160μl of CV solution (0.1%) for 5min, and the excess stain was eliminated by two washes with tap water. For solubilizing the bound CV, 160μl of acetic acid (33%) was added to the wells, and the optical absorbance was determined at 595nm using a FLUOstar Omega microplate reader.

In this study, the nature of the interaction of the combinational agents was evaluated by determination of the anti-biofilm capacity of the agents and using the fractional biofilm inhibitory concentration (FBIC). For calculating FBIC index, the observing equation was used: ΣFBIC=FBIC A+FBIC B=(MBIC of drug A in the combination/MBIC of drug A alone)+(MBIC of drug B in the combination/MBIC of drug B alone; Abdi Ali et al., 2015; Feldman et al., 2020). The lowest ΣFBIC index was chosen for the strongest interaction between two agents. The effect of two agents is considered as synergy when the ΣFBIC index is 0.5 or less, additive when the ΣFBIC index is between 0.5 and 1, indifferent when the ΣFBIC index is between 1 and 4, and antagonistic when the ΣFBIC index is 4 or more (Ramezani Ali Akbari and Abdi Ali, 2017; Feldman et al., 2020). Synergy means the interaction or cooperation of two or more organizations, substances, or other agents to produce a combined effect greater than the sum of their separate effects. Additive means the overall consequence, which is the result of two agents acting together and which is the simple sum of the effects of the agents acting independently. Indifferent means the combination has no increase in inhibitory activity of both agents. Antagonistic means the effect produced by the contrasting actions of two (or more) agents (Pillai et al., 2005).

To assess the effectivity of the most optimal combination (COS and CLI) on bacterial growth inhibition, the supernatant of the combination of treatments that acquired the best inhibitory effect and the lowest ΣFBIC was collected and grown on blood agar plates. One loopful (approximately 10μl) of the corresponding well was transferred into a separate blood agar plate. The transferred amount was evenly spread over the surface and the agar plate was incubated at 37°C for 24h. The bacterial growth of the combinational treatment was optically compared with the growth of bacteria with each agent separately as well as with the positive control (without treatment).

### Statistical Analysis

Data were reported as mean values ± SEM of at least three independent experiments (*n*=3) routinely performed in triplicate (three wells/condition). Results were analyzed using Prism 8.0 GraphPad Software (GraphPad, San Diego, CA, United States). Statistical significance was determined using ANOVA followed by Bonferroni *post-hoc* test. Differences were considered as statistically significant when *p*<0.05.

## Results

### AOS and COS Differentially Affect *S. aureus* and GBS V Growth

**Figure 2:**
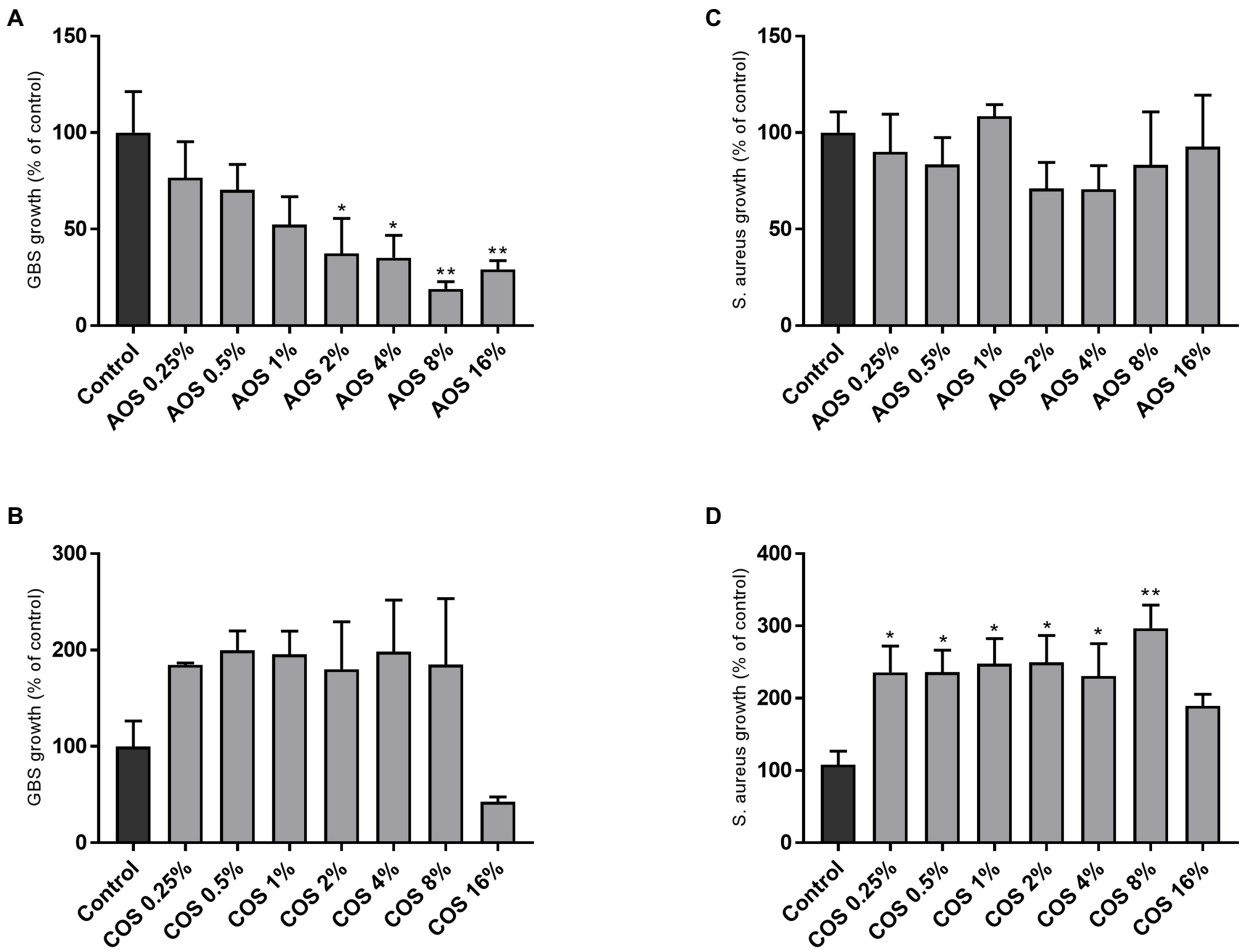
Effect of AOS and COS on bacterial growth of GBS V and *S. aureus* wood 46 strains in TSB. In order to identify the MIC of AOS and COS against GBS V **(A,B)** and *S. aureus* wood 46 **(C,D)**, seven 2-fold serial dilutions of each NDO were examined, as described in the Material and Methods section. Control represents the percentage of the maximum growth of bacteria without any intervention. Results are expressed as the percentage of bacterial growth (relative to control) as mean±SEM of three independent experiments each performed in triplicate. Statistical differences ^*^(*p*<0.05) and ^**^(*p*<0.01) compared to positive control were obtained using one-way ANOVA test. AOS, alginate oligosaccharides and COS, chitosan oligosaccharides.

Το evaluate whether AOS and COS can inhibit the growth of the two pathogenic strains, GBS V and *S. aureus* wood 46, bacterial growth in TSB in the absence and presence of increasing concentrations of AOS and COS was investigated. As shown in Figure 2, the addition of AOS caused a significant concentration-dependent reduction in the growth of GBS V. At AOS concentrations below 2% (0.25, 0.5, and 1%) no significant inhibition of GBS V growth was observed (Figure 2A). Although higher AOS concentrations decreased the growth of GBS V, the minimum inhibitory concentration was not identified, since the growth inhibition did not reach 90% or higher. Maximum inhibition of growth (81%) was achieved at concentration of 8% AOS. Unlike AOS, COS treatment (0.25–8%) did not inhibit, but even enhanced GBS V growth by up to 2-fold. Using the highest COS concentration (16%), a slight but not significant reduction in GBS V growth was measured (Figure 2B). Similar measurements of the effects of AOS and COS on the growth of *S. aureus* demonstrated unaltered growth of *S. aureus* in the presence of AOS (Figure 2C), whereas COS (0.25–8%) again caused an increase in bacterial (*S. aureus*) growth (Figure 2D).

### AOS Differentially Affect the Sensitization of GBS V Toward Four Different Antibiotics (AMP, CLI, TET, and TMP)

**Figure 3:**
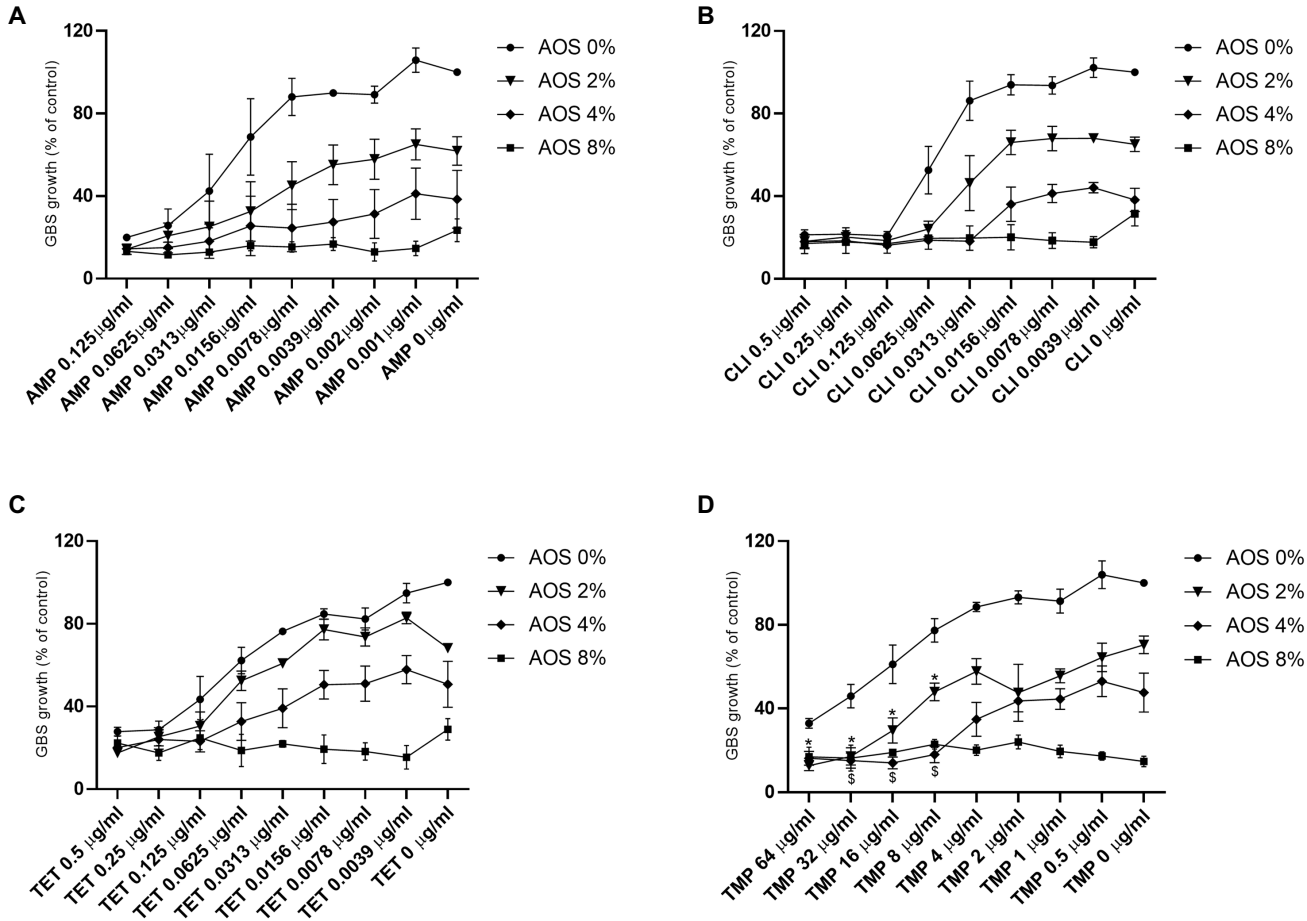
Effect of AOS in combination with AMP, CLI, TET and TMP on the growth of GBS V strain. To examine whether AOS has the feasibility to sensitize GBS V to antibiotics, a sensitization assay was performed as described in the material and method section. AOS (2, 4 and 8%) were combined with different concentrations of AMP **(A)**, CLI **(B)**, TET **(C)** and TMP **(D)**. Sensitization was only achieved when 2 and 4% AOS were combined with TMP. Star (for 2% AOS) or dollar (for 4% AOS) are representing a significant reduction of the combinational treatments comparing with both corresponding antimicrobial agents (AOS and antibiotics). Positive control represents the percentage of the absolute growth of bacteria (100% growth) without the presence of any treatment. The results are expressed as the percentage of bacterial growth as mean±SEM of three independent experiments each performed in a minimum of three replicates. Statistical differences *, ^$^ (*p*<0.05) compared to control and the corresponding concentrations of antibiotics and AOS, were obtained using a two-way ANOVA test.

In order to investigate whether AOS is able to sensitize GBS V to antibiotics (AMP, CLI, TET, and TMP), an antibiotic sensitization assay was conducted. Hereto, GBS V growth in the absence and presence of increasing concentrations of AOS and antibiotics was followed over time. A downward trend in GBS V growth is observed by all the AOS-antibiotic combinations tested, however, AOS-AMP and AOS-TET did not provoke any GBS sensitization to the antimicrobial agents, since any of combinational concentrations did not significantly decrease the bacterial growth comparing with corresponding concentration of AOS as well as with the antibiotic (Figures 3A,C). Interestingly, a striking reduction in the minimum inhibitory concentration of CLI was visible when AOS was combined. When 4% AOS was added to 0.0313μg/ml CLI, the same reduction of GBS growth was observed as obtained with three times higher CLI concentration (0.125μg/ml) without AOS supplementation (Figure 3B). Furthermore, AOS sensitized GBS V to the highest resistance concentrations of TMP (8–64μg/ml; Figure 3D). More specifically, 2 and 4% AOS induced a significant reduction of TMP at concentrations more than 8μg/ml, and from 8μg/ml to 32μg/ml, respectively (Supplementary Figure 1).

### AOS and COS Induce Different Changes in Biofilm Formation by GBS and *S. aureus*

**Figure 4:**
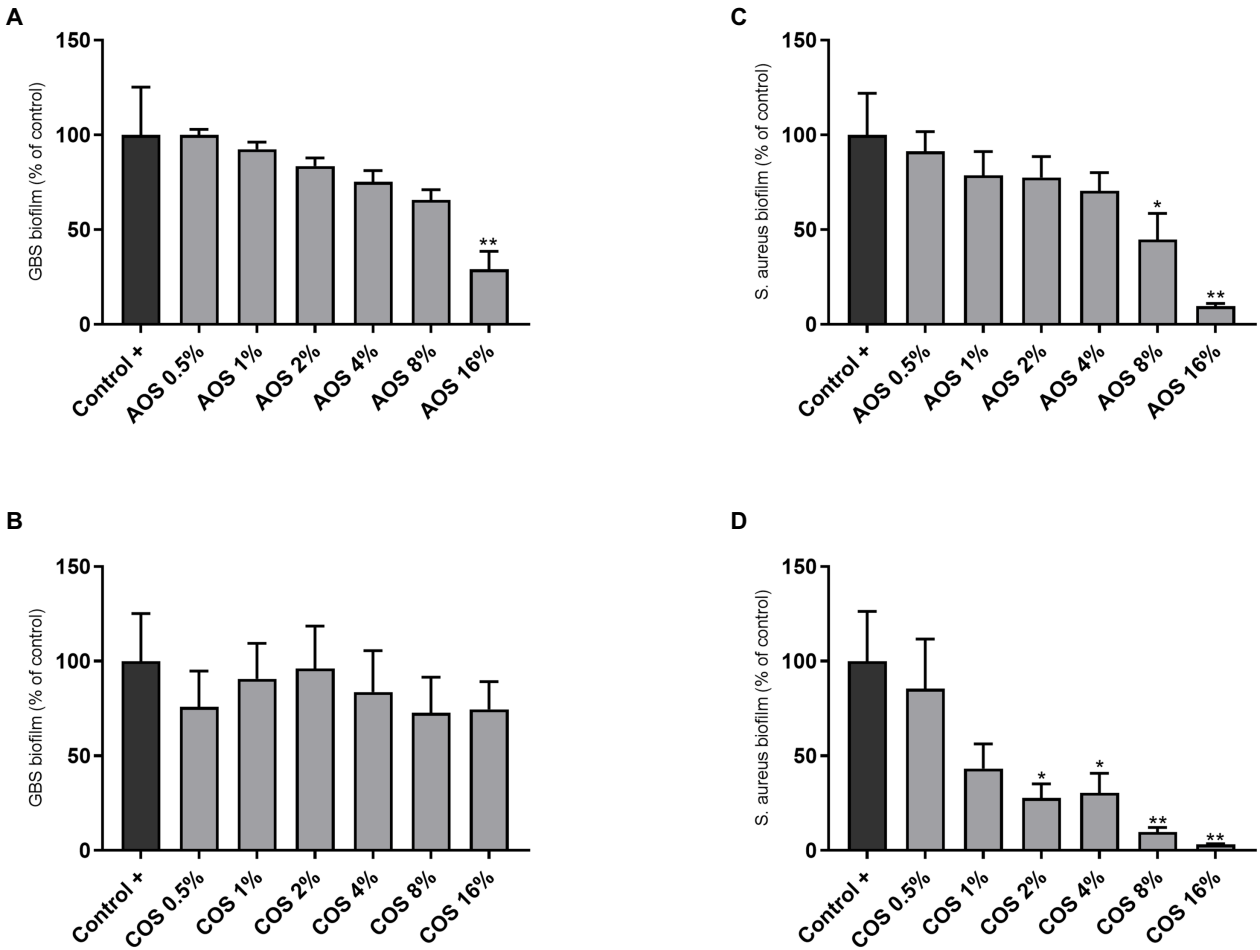
Anti-biofilm activity of AOS and COS against GBS V and *S. aureus* wood 46 strains. For the biofilm formation assay, six 2-fold serial dilutions of AOS and COS were tested after 24h of exposure, targeting the MBIC against GBS V **(A,B)** and *S. aureus* wood 46 **(C,D)**, as described in the Material and Methods section. Control (−) represents the negative control (uninoculated culture media without NDO treatment) and control (+) represents full-formed biofilms without any additional treatment 16% AOS and 8% COS are considered as MBIC against *S. aureus* wood 46. Results are expressed as the percentage of biofilm formation (relative to positive control) as mean±SEM of three independent experiments each performed in triplicate. Statistical differences ^*^(*p*<0.05) and ^**^(*p*<0.01) compared to positive control were obtained using one-way ANOVA test. AOS, alginate oligosaccharides and COS, chitosan oligosaccharides.

To assess the behavior of AOS and COS during biofilm formation, the biofilm-forming capacity of GBS V and *S. aureus* was conducted in presence and absence of AOS or COS by staining with CV. As shown in Figure 4A, the highest concentration of AOS (16%) induced a significant reduction (71%) of biofilm formation by GBS V compared to the positive control (without treatment). This cannot be considered as MBIC since this inhibition did not reach 90% or higher. COS treatment did not affect the GBS V biofilm formation (Figure 4B). In contrast, both AOS and COS showed an inhibitory effect on *S. aureus* biofilm formation. AOS showed an inhibitory effect in a concentration-dependent manner, although only at 8% AOS and 16% AOS the effects were statistically significant, as depicted in Figure 4C. With the highest AOS concentration (16%) even a 90% inhibition of biofilm formation was achieved (MBIC). COS (2–16%) treatment significantly reduced the biofilm formation of *S. aureus* wood 46. Precisely, COS 16% and COS 8% (MBIC) exhibited 97 and 90% inhibition of *S. aureus* biofilm formation, respectively. At concentrations below 2% COS no effect was observed (Figure 4D).

### Differential Interactions of COS and AOS With Five Different Antibiotics (AMP, CLI, CIP, TET, and TMP) Against Biofilm Formation of *S. aureus*

**Figure 5:**
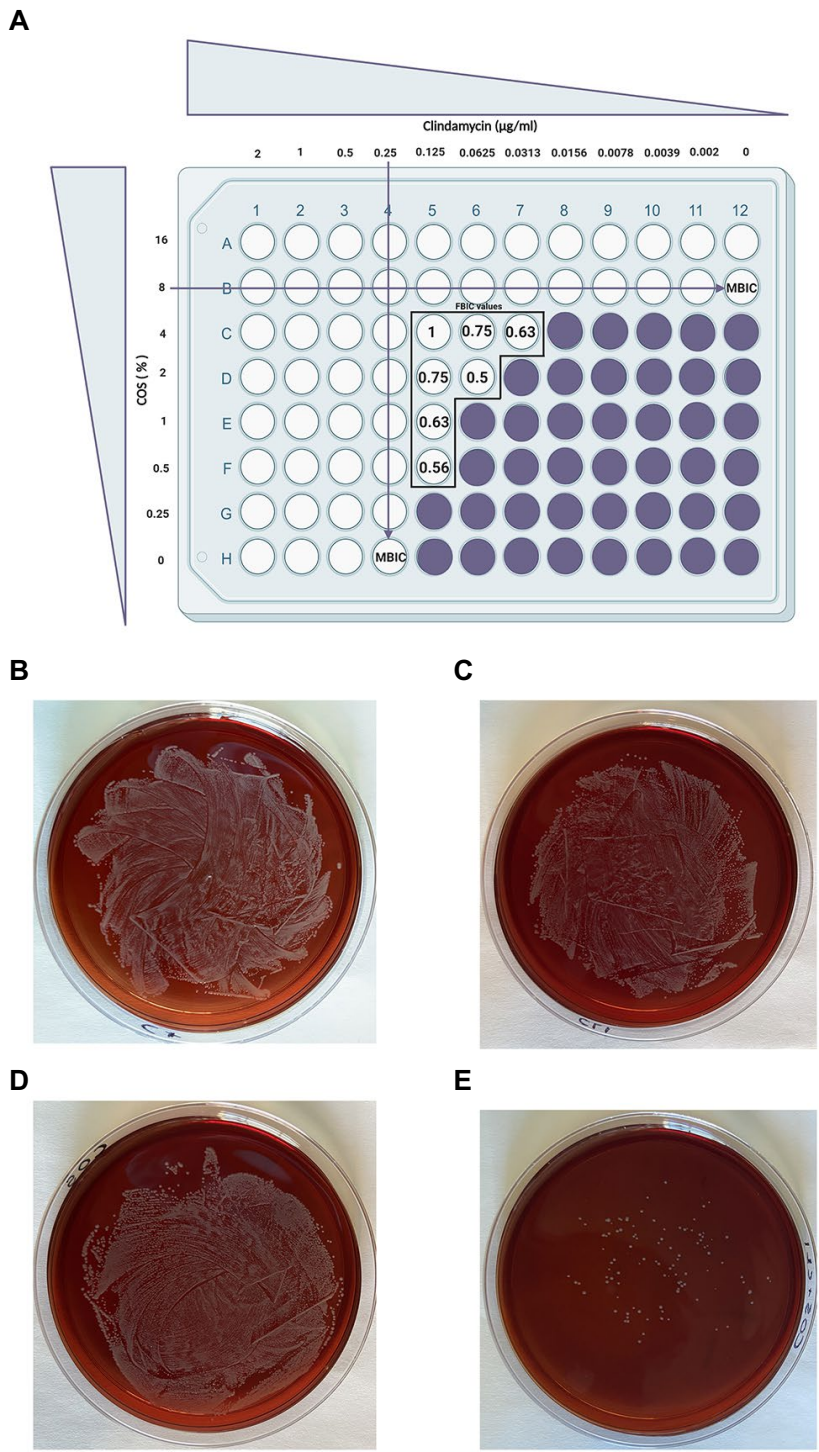
A simulated checkerboard assay for the combination of COS with CLI against biofilm formation of *S. aureus* and the subsequent growth of the synergistic combination on blood agar plates. As depicted in (**A**; Pillai et al., 2005), both COS and CLI were 2-fold serial diluted starting from 16% COS (2×MBIC) and 2μg/ml CLI (8×MBIC). Wells without color refer to full inhibition of *S. aureus* biofilm formation (≥ 90% biofilm inhibition) while in purple wells the *S. aureus* biofilm formation was not fully inhibited. The FBIC values of the wells that showed synergistic or additive effect were calculated as described in the material and methods section using the following equation: ΣFBIC=FBIC A+FBIC B=(MBIC of drug A in the combination/MBIC of drug A alone)+(MBIC of drug B in the combination/MBIC of drug B alone) and depicted in the figure for each well separately. Partial synergy was identified when 2% COS and 0.0625μg/ml CLI were combined with a corresponding FBIC value of 0.5. This specific FBIC value is considered as the lowest FBIC index among the combination wells that had full biofilm formation inhibition. The far-right column represents the control of COS treatment (without CLI treatment) while the bottom row, in which COS is absent, represents the control of CLI treatment. Finally, the well on the bottom right corner, in which both COS and CLI are absent, is used as positive control for biofilm production. The combination of the antimicrobial agents that provoke the synergistic effect, 2% COS with 0.0625μg/ml CLI were grown on blood agar FIGURE 2plates for 24h in order to determine whether the observed effect was attributed to their abilities to reduce the number of bacteria **(E)**. To identify the outcome of their interaction on bacterial growth, each agent was also grown separately, positive control which represents the absolute growth of bacteria without the presence of any treatment **(B)**, CLI 0.0625μg/ml **(C)** and COS 2% **(D)**. Finally, on a separate plate, the supernatant of the well without any intervention was grown, representing the positive control.

To test the possible additive effects of NDOs on the action of conventional antibiotics, a biofilm formation checkerboard assay was performed (Table 2). The different interactions were measured using the FBIC index as described in the material and methods section. Among different combinations tested, COS with CLI obtained a synergistic anti-biofilm effect. As shown in Figure 5A, a simulation of the checkerboard biofilm assay is depicted, demonstrating the synergistic effect of 2% COS in combination with 0.0625μg/ml CLI (FBIC value, 0.5) on *S. aureus* biofilm formation. This specific combination resulted in the lowest FBIC index among the different combinations that exhibited full inhibition of *S. aureus* biofilm formation. Along with the FBIC value related to synergy, the FBIC values of the wells, in which an additive effect was reported, were also calculated and mentioned in Figure 5A. Concerning the other antibiotics, an additive effect was reported with the treatments of AOS and COS in conjunction with TET, and the FBIC values were 0.54 and 0.75, respectively. Additive interaction was also observed when AOS was combined with CLI (FBIC value, 0.73). The nature of interaction of both AOS and COS with CIP (FBIC value, 1.01 and 1.03, respectively) or TMP (FBIC value, 1.01 for both) was characterized as indifferent. Finally, antagonism interaction was reported when AMP was combined with COS (FBIC value>4) and indifference when combined with AOS (FBIC value, 1.42).

**Table 2:** MBIC values, FBIC index and the nature of interaction between AOS and COS with AMP, CIP, CLI, TET, and TMP against *S. aureus* wood 46.

	COS				AOS			
Antibiotics (ATB)	ATB MBIC with COS (μg/ml)	COS MBIC with ATB (%)	FBIC	Interaction	ATB MBIC with AOS (μg/ml)	AOS MBIC with ATB (%)	FBIC	Interaction
AMP	0.128	0.25	>4	Antagonist	0.032	0.25	1.42	Indifferent
CIP	0.5	0.25	1.03	Indifferent	0.5	0.25	1.01	Indifferent
CLI	0.0625	2	0.5	Synergy	0.125	1	0.73	Additive
TET	0.125	1	0.75	Additive	0.125	0.5	0.54	Additive
TMP	1	4	1.01	Indifferent	1	8	1.01	Indifferent

Furthermore, the supernatant of the combinational treatment that achieved the most optimal effect (COS with CLI, FBIC value, 0.5) was collected and cultured on blood agar plates in order to identify whether the two agents could also reduce the bacterial growth in addition to the full inhibition of *S. aureus* biofilm formation. As depicted in Figure 5E, the *S. aureus* colonies treated with the COS-CLI combination were almost eliminated in comparison with the number of *S. aureus* bacteria in positive control group (Figure 5B), treated with 0.0625μg/ml CLI (Figure 5C) or 2% COS (Figure 5D).

## Discussion

In the present study, the anti-growth and anti-biofilm activities of AOS and COS against two pathogenic bacterial strains, GBS V and *S. aureus* wood 46, were evaluated. Furthermore, their combination with different antibiotics was tested to determine whether NDOs could enhance the function of antibiotics.

The antibacterial data revealed that AOS induce a strong inhibitory effect on the growth of GBS V, even at a low concentration (AOS 1%). One possible interpretation of such an effect is the anionic nature of AOS. Craft et al. (2019) showed that sialylated HMOs, which are negatively charged at homeostatic pH due to the sialic acid residues, exert antimicrobial activity against GBS III and Ia strains. In addition, the same group presented in another study that neutral fucosylated HMOs (2’-FL) did not have any substantial activity (Craft and Townsend, 2019). Despite the fact that the negative charge is assumed to play a substantial role in the antimicrobial abilities of AOS, further investigations are needed to confirm this mechanism of action. AOS significantly reduced the growth of *S. aureus* wood 46, and therefore may act in a strain-dependent manner. Interestingly, a depolymerized product of alginate (a mannuronic acid derivative) demonstrated an inhibition and high inhibitory activity against *S. aureus* (Hu et al., 2005). The differences in the anti-pathogenic effects of AOS might be related to the match or mismatch between the structural features of AOS (negatively charged) and the strain-specific bacterial target structures.

While COS increased the growth of both bacterial strains, this increase was only significant in the case of *S. aureus* wood 46. Concerning the observed bacterial growth, it can be hypothesized that instead of exerting antimicrobial effects, COS was utilized as a beneficial source for the growth and survival of GBS and *S. aureus*. These results concur with an earlier study (Yildirim-Aksoy et al., 2019), in which chitosan exhibit the capacity to stimulate growth. This could be related to the positively charged amino groups of chitosan, which bind to surface components and cell debris instead of cell surfaces of the related pathogen (Yildirim-Aksoy et al., 2019). Moreover, it is possible that GBS could reproduce and utilize degraded chitosan as the sole carbon source to benefit their growth (Yildirim-Aksoy et al., 2019). These observations were in contrast with previous reports indicating that the antimicrobial activities of COS might relate to the interaction between the positively-charged COS and the negatively-charged membrane residues (e.g., carbohydrate, proteins, and lipids; Jeon et al., 2001; Zheng and Zhu, 2003; Moon et al., 2007; Lin et al., 2009; Benhabiles et al., 2012). This effect can lead to cytoplasmic leakage and subsequently to cell death (Liu et al., 2004). For example, Benhabiles et al. (2012), confirmed the inhibitory effects of N-acetyl COS (NAc-COS) and COS on the growth of *S. aureus* ATCC 25923 and *S. aureus* ATCC 43300. COS with a higher molecular weight (MW; MW≥10kDa) are more effective in inhibition of different microorganisms, such as *S. aureus*, compared to fractions with lower MW (Jeon et al., 2001). These discrepancies might be attributable to methodological and experimental differences such as bacterial strains and structural characteristics of COS. In this regard, it has previously been shown that the antibacterial effect of COS is greatly dependent on their degree of polymerization or their MW (Jeon and Kim, 2000).

The potentiation of antibiotic activity in the therapy of multidrug-resistant organisms is a major goal of an anti-infective cure. In the present study, we investigated the ability of AOS to potentiate the activity of conventional antibiotics against the GBS V strain. AOS was selected since it exhibited the capacity to inhibit GBS V. Results obtained from the antibiotic sensitization assay indicated that AOS (2 and 4%) sensitizes GBS V to TMP by significantly decreasing the effective TMP concentration to observe a similar effect as obtained with more than eight times higher TMP concentration. Although sensitization of GBS V occurred only in the case of TMP, 4% AOS decreased the lowest effective concentration of CLI up to 4-fold (from 0.125 to 0.0313μg/ml CLI). Overall, a downward trend is observed in the growth of GBS V from all the combinations tested, however, this trend is mostly attributed to the effectivity of AOS starting from 8% AOS with the most potent anti-growth ability observed with 4 and 2% AOS. Therefore, these insights into the effects of AOS might provide new opportunities to develop treatments of GBS-associated infections for which effective treatment is currently extremely limited.

Biofilms are one of the most challenging resistant mechanisms of bacteria that secrete various enzymes and virulence factors (Graf et al., 2019). Different mechanisms related to the inhibition of biofilm formation are modification of cell-surface charge, inhibition of bacterial growth, and prevention of microbial adhesion (Roy et al., 2018).

In our study, AOS significantly reduced the biofilm formation of both GBS V (4, 8, and 16% AOS) and *S. aureus* wood 46 (8 and 16% AOS). So far, anti-biofilm activities of AOS were only identified against Gram-negative bacteria such as *P. aeruginosa* (Powell et al., 2013, 2018; He et al., 2014). To the best of our knowledge, this is the first study indicating the anti-biofilm effects of AOS against gram-positive bacteria. We hypothesized that the negative charges of AOS interact with positively charged components of the biofilm matrix. Indeed, concerning staphylococci biofilms, one of the most important positively charged polymers is called as polysaccharide intercellular adhesin (PIA). PIA is involved in at least the majority of the staphylococcal biofilm-associated infections and constitutes the main molecule responsible for intercellular adhesion. The cationic PIA polymer (at neutral or basic pH) interacts with the negatively charged bacterial cell surface (e.g., with negatively charged teichoic acids) through multivalent electrostatic interactions (Arciola et al., 2015). Since PIA plays such a significant role in staphylococci biofilm formation, its inhibition by AOS could lead to obstructing the adhesive role of PIA and consequently inhibiting biofilm formation. There is limited information available related to the formation of streptococci biofilms such as the composition of the GBS biofilm matrix. However, recent studies showed that this type of biofilm is mainly composed of proteins and eDNA, while polysaccharides represent a minor proportion (D’Urzo et al., 2014; Alvim et al., 2019). The anti-biofilm effect of AOS against GBS V can be attributed to the disruption of the intramolecular interactions in EPS that might occur due to the negative charge of AOS. These assumptions are in line with the results found by Taylor Nordgård and Draget (2011), who reported that alginates can disrupt intramolecular interaction in EPS (e.g., mucus), and competitively inhibit the interpolymer cross-links, weakening the biofilm structures. Anti-biofilm potential of AOS on the biofilms of GBS has not been identified so far. Therefore, further research is needed to identify the underlying mechanisms.

In contrast with AOS, the effect of COS on biofilm formation differs among the two bacterial species. COS treatment was ineffective against biofilm formation of GBS V, which is in line with the antibacterial data of COS in this study. On the other hand, there was a strong anti-biofilm inhibitory effect of different COS concentration (2, 4, 8, and 16% COS) against *S. aureus* wood 46. Interestingly, the strong effect of COS against the biofilm formation of *S. aureus* wood 46 is in contrast with the observed (non-existing) antibacterial effect since a significant increase in bacterial growth was observed. This contradiction might be attributed to the interference of COS with the compartments of the biofilm matrix that leads to the inhibition of biofilm formation, instead of adherence to the bacterial surface that has been previously proved to cause growth reduction (Felipe et al., 2019). Our results supported the finding that chitosan displays an anti-biofilm activity against *S. aureus* strains of bovine origin (Asli et al., 2017). Additionally, a significant inhibitory effect of LMW chitosan on *S. aureus* V329 biofilm formation was demonstrated (Felipe et al., 2019). Anti-biofilm properties of COS most likely rely on the polycationic nature due to its protonated amino groups, which interact electrostatically with the negatively charged biofilm components (e.g., proteins and eDNA; Khan et al., 2020). Hence, this electrostatic interaction may inhibit the formation of biofilm (Jiang et al., 2014). The observed inhibitory effect of COS on biofilm formation of *S. aureus* wood 46 in the current study can be attributed to the prevention of biofilm formation rather than on the destruction of preformed biofilms, since no changes were observed when COS was added for 24h after formation of established biofilms (Supplementary Figure 2A). Therefore, it can be hypothesized that COS interferes biofilm formation and development in early stages.

**Figure 6:**
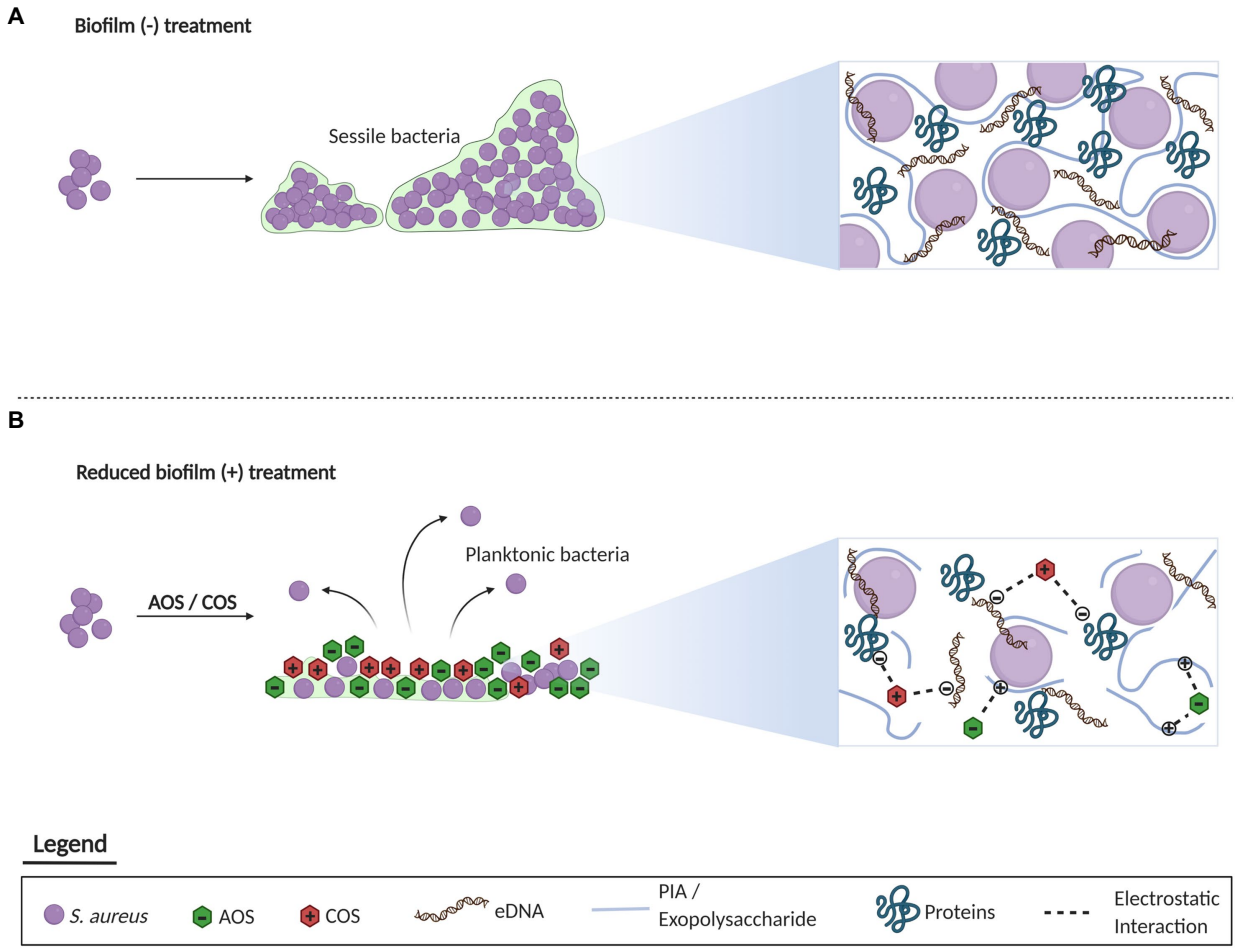
Representation of the proposed mechanism underlying the inhibitory effect of AOS and COS on biofilm formation of *S. aureus*. Concerning the untreated biofilm, a mature biofilm is formed by *S. aureus* in the absence of any treatment mainly composed of PIA, eDNA, and proteins **(A)**. On the other hand, administration of AOS and COS may inhibit the formation of biofilm through the electrostatic interaction of oligosaccharides with the charged components of the extracellular matrix **(B)**. This figure was created with BioRender.com.

In summary, based on the anti-biofilm effects displayed by AOS and COS against *S. aureus* wood 46, a proposed mechanism of action is schematized in Figure 6. In comparison with the untreated bacteria that are able to form a mature biofilm, biofilm formation with treatment of AOS and COS is inhibited. This inhibitory anti-biofilm formation might be attributed to the charge of those two NDOs. AOS might electrostatically interact with the positively charged PIA while COS might interact with the negatively charged proteins and the eDNA of the extracellular matrix.

In support of this assumption: neutral fructo-oligosaccharides did not exert any anti-biofilm activity against *S. aureus* wood 46 (Supplementary Figure 2B).

The development of bacterial resistance is one of the major concerns nowadays that affects the efficacy of several antibiotics for the treatment of severe infections. Combination therapy is considered as an effective approach to increase the potency of existing antibiotics and thereby combat antimicrobial resistance.

First, a checkerboard biofilm formation assay was performed testing the nature of the interactions of AOS and COS with five common-used antibiotics against *S. aureus* wood 46. The combination of COS and AOS with several antibiotics was investigated before, however, these studies was mainly focused on the reduction of bacterial growth rather than on the inhibition of biofilm formation (Tin et al., 2009; Khan et al., 2012; Asli et al., 2017; Kim et al., 2017). In the present study, the combination of COS with CLI and TET (targeting ribosomal protein 50S and 30S subunits, respectively) showed synergistic and additive activity against *S. aureus* wood 46 biofilm formation, respectively. Additive interaction also occurred when AOS was combined with both antibiotics. COS can inhibit the biofilm formation through ionic interactions (Khan et al., 2020), therefore it can enhance the accumulation of CLI into the biofilm and inhibition of protein synthesis, showing a synergistic effect. Since CLI inhibits bacterial protein synthesis by binding to the 50S subunit of the bacterial rRNA inside the cell, it is expected that it can also reduce exoprotein production in *S. aureus* biofilms. Hu et al. (2019) studied the effects of sub-inhibitory CLI on the production of *S. aureus* exoproteins and demonstrated that subinhibitory concentrations of CLI considerably decrease the *S. aureus* biofilm exoprotein. The additive effect observed by the interaction of AOS and COS with TET might be attributed to a similar mechanism of action, as TET also inhibits protein synthesis, although binding to the 30S subunit.

Interestingly, two bactericidal antibiotics (AMP and CIP) used in this study showed indifferent or antagonistic effects in combination with AOS and COS. Yang et al. (2015) demonstrated that two bactericidal antibiotics (vancomycin and CIP) were tested on *Staphylococcus epidermidis* biofilms and found that the combinational treatment with these two antibiotics can reduce the efficacy of these individual treatments. These observations might be related to the formation of persister cells induced by stress from antibiotics (Dörr et al., 2010). Moreover, the antagonistic effect deriving from the combination of COS with AMP can be partly explained by the fact that both agents target extracellular cell compartments. So, the positively charged group of COS can bind to the negatively charged compartments of the cell membrane, leading to the death of bacteria (Liu et al., 2004), while AMP inhibits cell wall synthesis (Epand et al., 2016), leading to competition among the two agents.

## Conclusion

In the present study, we have shown that AOS and COS modulate both bacterial growth and biofilm production of GBS V and *S. aureus* wood 46, respectively. In addition to the observed anti-growth and anti-biofilm properties of both NDOs, the anti-biofilm and anti-growth potential in combination with different antibiotics was evaluated. The synergistic effect of COS with CLI against *S. aureus* and the ability of AOS to sensitize GBS V to TMP were the most promising results offering new perspectives to help combat antimicrobial resistance. Given the increasing need for antimicrobial alternatives and the capability of NDOs to serve as antibacterial agents, future efforts should focus on assessing the antimicrobial effects of AOS and COS against additional species of both Gram-positive and Gram-negative pathogens and evaluating combination therapy with different antibiotics. Moreover, investigating the mechanisms underlying the antimicrobial, and anti-biofilm capacity of NDOs, as well as the interaction with antibiotics is necessary to further establish the therapeutic potential of NDOs.

## Data Availability Statement

The original contributions presented in the study are included in the article/Supplementary Material, further inquiries can be directed to the corresponding author.

## Author Contributions

MA, GF, RP, JP, and SB: conceptualization. MA and G-NI: methodology, software, investigation, data curation, writing – original draft preparation, and visualization. MA, G-NI, and SB: validation. MA: formal analysis. JP and GF: resources. JP, SB, GF, and RP: writing – review and editing. GF and SB: supervision. GF, RP, JP, and SB: project administration. GF, SB, and RP: funding acquisition. All authors have read and agreed to the published version of the manuscript. All authors contributed to the article and approved the submitted version.

## Conflict of Interest

The authors declare that the research was conducted in the absence of any commercial or financial relationships that could be construed as a potential conflict of interest.

## Publisher’s Note

All claims expressed in this article are solely those of the authors and do not necessarily represent those of their affiliated organizations, or those of the publisher, the editors and the reviewers. Any product that may be evaluated in this article, or claim that may be made by its manufacturer, is not guaranteed or endorsed by the publisher.

## Figures and Tables

**Table:** 

AMP	Ampicillin
AOS	Alginate oligosaccharides
ATB	Antibiotics
BM	Biofilm medium
CIP	Ciprofloxacin
CLI	Clindamycin
COS	Chitosan oligosaccharides
CV	Crystal violet
eDNA	Extracellular DNA
EPS	Extracellular polymeric substances
FBIC	Fractional biofilm inhibitory concentration
GBS	Group B *Streptococcus*
HMOs	Human milk oligosaccharides
MBIC	Minimum biofilm inhibitory concentration
MIC	Minimum inhibitory concentration
MW	Molecular weight
NAc-COS	N-acetyl COS
NDOs	Non-digestible oligosaccharides
OD	Optical density
PBS	Phosphate-buffered saline
PIA	Polysaccharide intercellular adhesin
*S. agalactiae*	*Streptococcus agalactiae*
*S. aureus*	*Staphylococcus aureus*
TET	Tetracycline
TMP	Trimethoprim
TSB	Tryptic soy broth
WBS	Working bacterial solution
