# Supplementary material for: Antimicrobial Activities of Alginate and Chitosan Oligosaccharides Against Staphylococcus aureus and Group B Streptococcus
Source: Front Microbiol. 2021 Sep 13;12:700605. doi: 10.3389/fmicb.2021.700605 (PMC8473942; doi:10.3389/fmicb.2021.700605)
Supplement: Supplementary file 1 [file Table_1.DOCX]

Supplementary Material

## Supplementary Figure 1


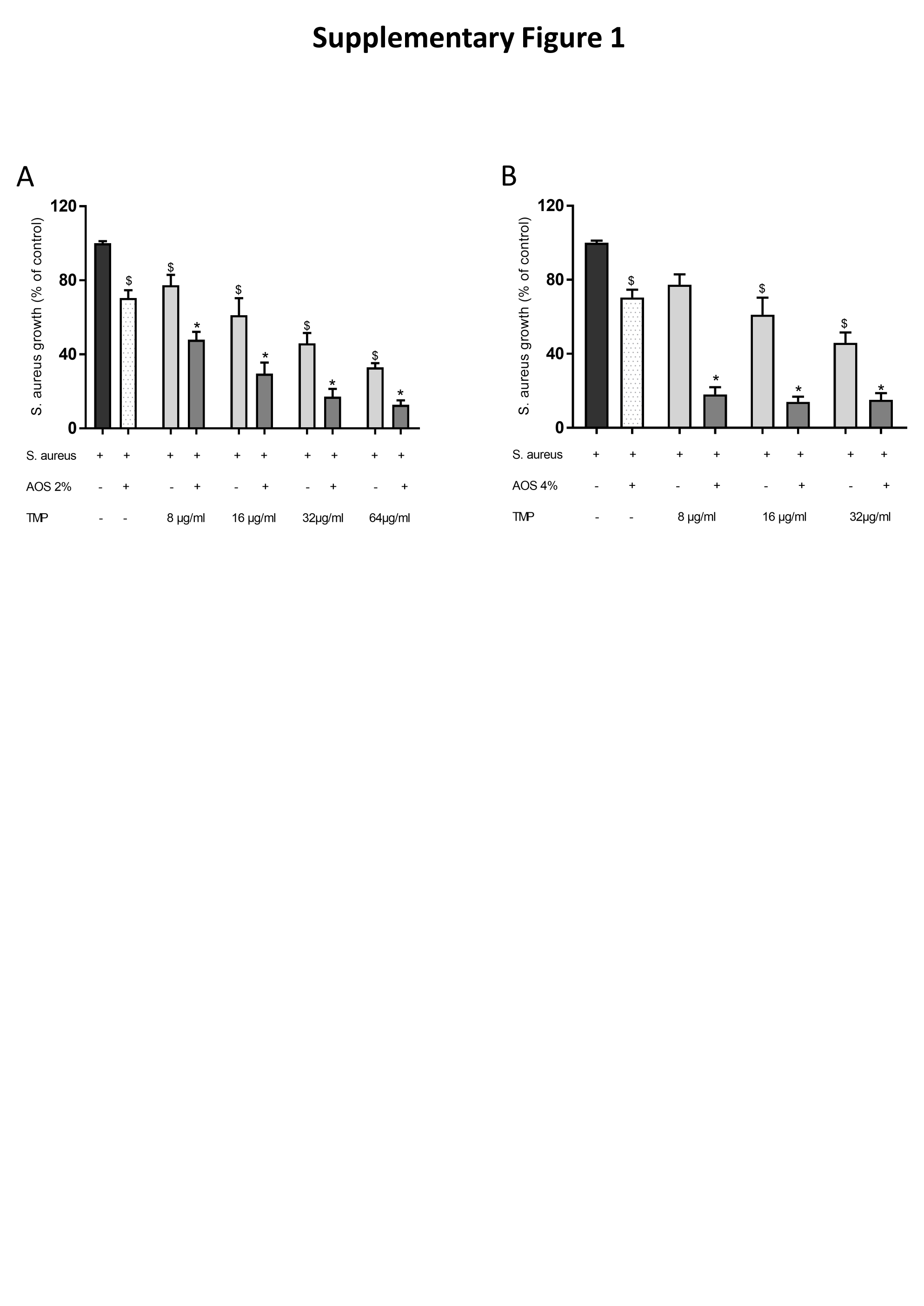


## Supplementary Figure 1

**Effective combination of 2% and 4% AOS and TMP on the growth of GBS.** To examine whether 2% and 4% AOS have the feasibility to sensitize GBS to TMP, a sensitization assay was performed as described in the material and method section. AOS (2%) were combined with different concentrations of TMP (**Supplementary figure 1A**) and AOS (4%) were combined with different concentrations of TMP (**Supplementary figure 1B**). Star (*) is representing a significant reduction (P < 0.05) of the combinational treatments comparing with corresponding antimicrobial agents (AOS and antibiotics) and positive control. Dollar ($) is representing a significant reduction (P < 0.05) of AOS or TMP groups comparing with positive control. Positive control represents the percentage of the absolute growth of bacteria (100% growth) without the presence of any treatment. The results are expressed as the percentage of bacterial growth as mean ± SEM of three independent experiments each performed in a minimum of 3 replicates. Two-way ANOVA test was used for statistical analysis.


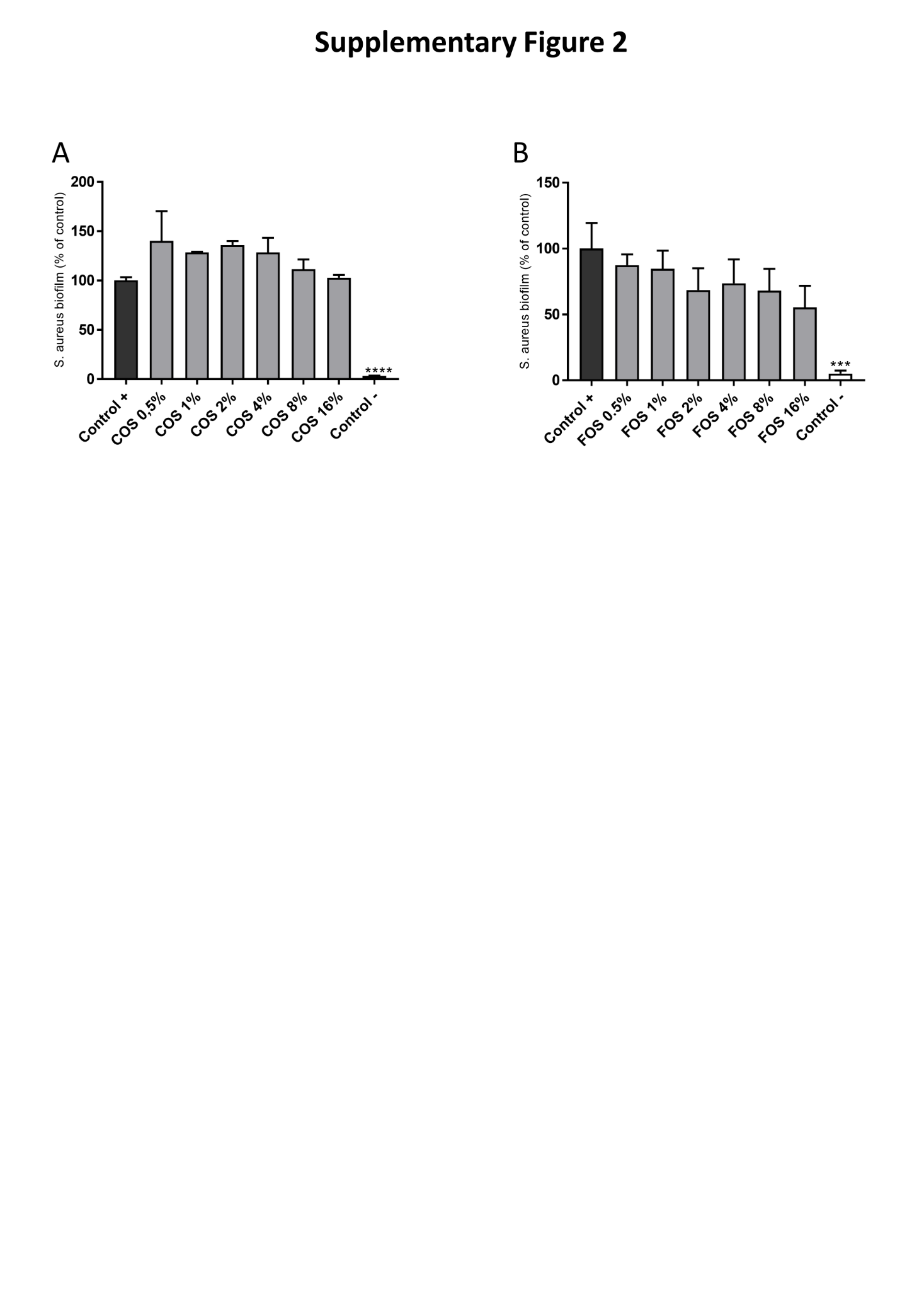


## Supplementary Figure 2

## Supplementary Figure 2

**Preventive effect of biofilm formation by FOS and anti-biofilm activity of COS against *S. aureus*.** For the biofilm formation assay, six different concentrations (0.5-16 %) of COS and FOS were tested for 24 h after formation of established biofilms (**Supplementary figure 2A**) and 24 h before formation of established biofilm (**Supplementary figure 2B**), respectively, as described in the material and methods section. Control (-) represents the negative control (uninoculated culture media without NDO treatment) and control (+) represents full-formed biofilms without any additional treatment. Results are expressed as the percentage of control (relative to positive control) as mean ± SEM of three independent experiments each performed in triplicate. Statistical differences *(P < 0.05), **(P < 0.01), ***(P < 0.001) and ****(P < 0.0001) compared to positive control were obtained using one-way ANOVA test.
